# The Enigmatic Role of Serum & Glucocorticoid Inducible Kinase 1 in the Endometrium

**DOI:** 10.3389/fcell.2020.556543

**Published:** 2020-10-21

**Authors:** Florian Lang, Janet Rajaxavier, Yogesh Singh, Sara Y. Brucker, Madhuri S. Salker

**Affiliations:** ^1^Department of Physiology, Eberhard-Karls University, Tübingen, Germany; ^2^Research Institute of Women’s Health, Eberhard-Karls University, Tübingen, Germany; ^3^Institute of Medical Genetics and Applied Genomics, Eberhard-Karls University, Tübingen, Germany

**Keywords:** SGK1, cell survival, cell migration, pregnancy, infertility

## Abstract

The serum- and glucocorticoid-inducible kinase 1 (SGK1) is subject to genetic up-regulation by diverse stimulators including glucocorticoids, mineralocorticoids, dehydration, ischemia, radiation and hyperosmotic shock. To become active, the expressed kinase requires phosphorylation, which is accomplished by PI3K/PDK1 and mTOR dependent signaling. SGK1 enhances the expression/activity of various transport proteins including Na^+^/K^+^-ATPase as well as ion-, glucose-, and amino acid- carriers in the plasma membrane. SGK1 can further up-regulate diverse ion channels, such as Na^+^-, Ca^2+^-, K^+^- and Cl^–^ channels. SGK1 regulates expression/activity of a wide variety of transcription factors (such as FKHRL1/Foxo3a, β-catenin, NFκB and p53). SGK1 thus contributes to the regulation of transport, glycolysis, angiogenesis, cell survival, immune regulation, cell migration, tissue fibrosis and tissue calcification. In this review we summarized the current findings that SGK1 plays a crucial function in the regulation of endometrial function. Specifically, it plays a dual role in the regulation of endometrial receptivity necessary for implantation and, subsequently in pregnancy maintenance. Furthermore, fetal programming of blood pressure regulation requires maternal SGK1. Underlying mechanisms are, however, still ill-defined and there is a substantial need for additional information to fully understand the role of SGK1 in the orchestration of embryo implantation, embryo survival and fetal programming.

## Introduction

Serum and glucocorticoid-inducible kinase 1 (SGK1) was discovered as an immediate early gene, transcriptionally induced in rat mammary cancer cells by glucocorticoids and serum (Webster et al., 1993). SGK1 is evolutionarily conserved and is found in almost all tissues (Arencibia et al., 2013). However, it is strictly transcriptionally and post-transcriptionally regulated (Waldegger et al., 1997; Brickley et al., 2002; Belova et al., 2006; Lang et al., 2006; Di Cristofano, 2017). Enhanced activity of SGK1 plays a decisive role in the pathophysiology of several clinical disorders, such as metabolic syndrome, fibrosis, vascular occlusion, tissue calcification, neurodegeneration, inflammation, autoimmune disease, malignancy and compromised female reproduction (Salker et al., 2011; Harries et al., 2012; Kleinewietfeld et al., 2013; Lang and Voelkl, 2013; Pelzl et al., 2017). Twin studies have disclosed a gain-of-function SGK1 gene variant in intron 6 [I6CC] and in exon 8 [E8CC/CT] (Busjahn et al., 2004). The prevalence of the I6C/E8CC/CT genotype was approximately 3–5% in Caucasians and 10% in African/Asian population (Busjahn et al., 2004; Schwab et al., 2008). Individuals with the SGK1 polymorphism were associated with moderately enhanced occurrence of hypertension, shortened QT interval in electrocardiograms, stroke, obesity and diabetes (Busjahn et al., 2004; Schwab et al., 2008). A recent paper also reported that SGK1 variants were associated with chronic heart disease and depression in the Chinese Han population (Han et al., 2019).

The present review provides a short synopsis of the mechanisms regulating SGK1 expression and activity, the molecular SGK1 targets as well as the SGK1-dependent cell functions and survival. We also highlight the role of SGK1 in obesity and diabetes, and in inflammatory diseases such as endometriosis. Most importantly, the review amplifies on the involvement of SGK1 in the regulation of embryo implantation, embryo survival and fetal programming.

## SGK1 Regulation

**S**erum- and **G**lucocorticoid- **k**inases (SGKs) are members of the AGC (PK**A**-, PKB-,PK**G**- and PK**C**-related) family of serine/threonine kinases (Lang et al., 2006; Heikamp et al., 2014; Di Cristofano, 2017). SGKs represent one of the most evolutionarily conserved groups of protein kinases in the eukaryotic kingdom (Heikamp et al., 2014). The SGK family consists of three distinct but highly homologous isoforms (SGK1, SGK2 and SGK3) that are produced from three distinct genes localized on different chromosomes (Kobayashi et al., 1999).

Structurally, SGK1 consists of three domains: an N-terminal variable region, a catalytic domain, and the C-terminal tail (Zhao et al., 2007). SGK1 is subject to rigorous temporal and spatial regulation, mostly *via* phosphorylation of two conserved residues; one in the activation loop and the other in the hydrophobic motif within the C-tail, which is indispensable for full activation (Kobayashi and Cohen, 1999; Biondi et al., 2001).

Expressed SGK1 protein is not constitutively active but requires activation to be functional (Di Cristofano, 2017). In 1999, two key studies reported that SGK1 phosphorylation and activation was governed by the PI3K signaling cascade (Kobayashi and Cohen, 1999; Park et al., 1999). These studies emerged from the concept that the catalytic and C-terminal domains of SGK1 are highly homologous to that of other AGC kinases (Kobayashi and Cohen, 1999; Park et al., 1999). SGK1 function is directly dependent on mammalian target of rapamycin (mTOR) phosphorylation. Following the mTOR-dependent hydrophobic motif (H-motif) phosphorylation on serine 422 (García-Martínez and Alessi, 2008), the kinase changes into an open conformation for phosphorylation and complete activation by 3-phosphoinositide-dependent kinase-1 (PDK1) (Hong et al., 2008).

A plenitude of studies has now shown that SGK1 transcription is regulated by a multitude of stimuli and several inhibitors (Table 1). Stimulators of SGK1 activity and signaling involved are listed in Table 2 (Kobayashi and Cohen, 1999; Biondi et al., 2001; Collins et al., 2003; Lang et al., 2006). SGK1 activity is suppressed by the phosphatase and tensin homolog (PTEN), which degrades PIP3 (Biondi et al., 2001).

**TABLE 1:** Regulators of SGK1 expression.

**Regulator**	**Example**	**References**
Stimulating cell stressors	cell shrinkage, dehydration, mechanic tear, oxidative stress, heat shock, UV radiation, DNA damage, ischemia, neuronal injury, neuronal excitation, enhanced glucose concentrations, advanced glycation end products (AGEs), peroxisome proliferator-activated receptor γ (PPARγ)	Leong et al., 2003; Saad et al., 2005; Lang and Stournaras, 2013; Lang and Voelkl, 2013; Lang et al., 2018
Stimulating hormones and mediators	glucocorticoids, mineralocorticoids, gonadotropins, progesterone, 1,25(OH)_2_D_3_, erythropoietin, morphine, transforming growth factor-β (TGF-β), interleukin-6, fibroblast and platelet-derived growth factor, thrombin, endothelin	Mizuno and Nishida, 2001; Lang and Stournaras, 2013; Lang et al., 2018.
Inhibitors	serum starvation, heparin, dietary iron, nucleosides, nephrilin, ageing	Lang and Stournaras, 2013; Lang et al., 2018. Harries et al., 2012; Lang et al., 2018.
Signaling regulating SGK1 transcription	cytosolic Ca^2+^, cyclic AMP, stress-activated protein kinase-2 (SAPK2 or p38 MAPK) protein kinase C (PKC), protein kinase RAF, extracellular signal-regulated kinase 1/2 (ERK1/2) and 5 (ERK5) phosphatidylinositide-3-kinase (PI3K), reactive oxygen species (ROS), NADPH oxidases, nitric oxide, EWS/NOR1(NR4A3) fusion protein	Mizuno and Nishida, 2001; Lang and Stournaras, 2013; Lang et al., 2018.
SGK1 promotor binding sites	glucocorticoid receptor (GR), mineralocorticoids receptor (MR), progesterone receptor (PR), 1,25(OH)_2_D_3_ receptor (VDR), Retinoids receptor (RXR), Farnesoids receptor (FXR), sterol regulatory element-binding protein (SREBP), PPARγ, cAMP response element-binding protein (CREB), p53 tumor suppressor protein, Sp1 transcription factor, activator protein 1 (AP-1), activating transcription factor 6 (ATF6), heat shock factor (HSF), reticuloendotheliosis viral oncogene homolog (c-Rel), nuclear factor kappa- B (NFκB), signal transducers and activators of transcription (STAT), TGF-β-dependent transcription factors SMAD3 and SMAD4, forkhead activin signal transducer (FAST), transcription factor TonE binding protein (TonEbP/NFAT5)	Lang and Stournaras, 2013; Lang et al., 2018.

**TABLE 2:** Regulators of SGK1 activity.

**Regulators**	**Example**	**References**
Stimulators of SGK1 activity	insulin, IGF1, hepatic growth factor (HGF), follicle stimulating hormone (FSH), thrombin, corticosterone, lithium, neuronal depolarization, oxidation, hypertonicity, fibronectin	Lang et al., 2006; Lang and Stournaras, 2013. Waldegger et al., 1997; Lee et al., 2012; Lang and Stournaras, 2013; Lang et al., 2018
Signaling of SGK1 activation	PI-3 kinase-sensitive 3-phosphoinositide (PIP3)-dependent kinases PDK1 and PDK2, Na^+^/H^+^ exchanger regulating factor 2 (NHERF2), WNK1 (lysine deficient protein kinase 1), mammalian target of rapamycin mTOR complex-2 (mTORC2) composed of mTOR, Rictor (rapamycin-insensitive companion of mTOR), Sin1 (stress-activated protein kinase-interacting protein 1), mLST8 and Protor-1, p38α MAPK, ERK5, cAMP, Ca^2+^-sensitive calmodulin-dependent protein kinase kinase (CaMKK), G-protein Rac1	Waldegger et al., 1997; Kobayashi et al., 1999; Lang et al., 2006; Dibble et al., 2009; Peterson et al., 2009; Rosner et al., 2009; Heise et al., 2010; Lyo et al., 2010; Treins et al., 2010; Pearce et al., 2011; Fang et al., 2012; Hall et al., 2012; Na et al., 2013; Thomanetz et al., 2013; Domhan et al., 2014; Tsai et al., 2014; Lang et al., 2018.

Within the first 60 amino acids are the residues required for SGK1 degradation (Brickley et al., 2002). More precisely, a six amino acid motif (deficient of lysines) is required for ubiquitination and degradation by the 26S proteasome (Bogusz et al., 2006). Degradation of SGK1 requires four different E3 ubiquitin ligases: U-box E3 ubiquitin ligase CHIP, transmembrane E3 ubiquitin ligase HRD1, ubiquitin ligase neural precursor cell expressed developmentally downregulated gene 4-like (NEDD4L) and as shown more recently, a complex that includes Rictor, Cullin-1 and Rbx1 (Zhou and Snyder, 2005; Arteaga et al., 2006; Belova et al., 2006; Gao et al., 2009). Inhibitors of SGK1 degradation include glucocorticoid-induced leucine zipper protein-1 (GILZ) (Soundararajan et al., 2010).

## SGK1 Targets

SGK1 phosphorylates serine/threonine at the following amino acid sequences: R-X-R-X-X-(S/T)-phi and R-R-X-S/T (X = any amino acid, R = arginine, phi = hydrophobic amino acid) (Park et al., 1999). Other SGK1 target proteins are similarly phosphorylated by other kinases, such as SGK2, SGK3 and protein kinase B (PKB/Akt) isoforms (Debonneville et al., 2001).

SGK1-regulated proteins are listed in Table 3. They include diverse ion channels, transporters and carriers (Lang et al., 2006). SGK1 regulates a number of signaling pathways and transcription factors including NFκB, CREB and FOXO3a (McCaig et al., 2011; Ohashi et al., 2011; Sahin et al., 2013; Voelkl et al., 2015). Interestingly, SGK1 can upregulate NFκB, by phosphorylation of the inhibitory protein IκB and downregulate NFκB by activation of N-Myc downstream-regulated gene-1 (NDRG1)–a negative regulator of NFκB signaling (Murakami et al., 2010; Zarrinpashneh et al., 2013). The only proteins hitherto known to be exclusive SGK1 targets are NDRG1 and NDRG2 (Weiler et al., 2013; Matschke et al., 2015).

**TABLE 3:** SGK1-regulated target proteins.

**Target**	**Example**	**References**
Stimulation of enzymes	ubiquitin ligase NEDD4-2, inducible nitric oxide synthase (iNOS), phosphomannose mutase 2 (PMM2), phosphatidylinositol-3-phosphate-5-kinase (PIKfyve), serine/threonine kinase WNK4, ERK2, mitogen-activated protein kinase/ERK kinase kinase 3 (MEKK3), stress-activated kinase (SEK1), B-Raf kinase, glycogen synthase kinase 3 (GSK-3), p53-ubiquitinating MDM2 Notch1-IC protein degrading Fbw7	Firestone et al., 2003; Lang and Stournaras, 2013
Stimulation of transcription factors	CREB, AP-1, p53 tumor suppressor protein, CBFA1, MSX2, SOX9	Terada et al., 2008; Rotte et al., 2011; Borst et al., 2012; Eylenstein et al., 2012; Lang and Stournaras, 2013; Firestone et al., 2003; Lang et al., 2006, 2018.
Inhibition of transcription factors	NFκB, p53, forkhead box O3a- protein (FOXO3a)	Murakami et al., 2010; Lang and Stournaras, 2013. Dehner et al., 2008; Lang and Stournaras, 2013; Sahin et al., 2013.
Ion channels	epithelial sodium channel (ENaC), voltage-gated Na^+^ channels (SCN5A), acid sensing (ASIC1) Na^+^ channels, renal outer medullary K^+^ channels (ROMK1) voltage-gated K^+^ channels KCNE1/KCNQ1, KCNQ4, voltage-gated K^+^ channels Kv1.3, Kv1.5, Kv7.2/3, Kv4.3, voltage-gated K^+^ channels hERG, Ca^2+^release-activated Ca^2+^ channel ORAI and its stimulator STIM, transient receptor potential channels (TRPV4, TRPV5, TRPV6), kainate receptor GluR6, unselective cation channel 4F2/LAT, ClCka/barttin Cl^–^ channels, ClC2 Cl^–^ channels, CFTR Cl^–^ channels, VSOAC Cl^–^ channels	Giebisch, 1998; Arteaga and Canessa, 2005; Seebohm et al., 2005; Lang et al., 2006; Lang and Shumilina, 2013.
Carriers and pumps	Na^+^,K^+^,2Cl^–^ cotransporters (NKCC2), NaCl cotransporter (NCC), Na^+^/H^+^ exchangers (NHE1, NHE3), facilitative (GLUT1, GLUT4), Na^+^-coupled (SGLT1) glucose transporters, amino acid (ASCT2, SN1, B(0)AT1, EAAT1, EAAT2, EAAT3, EAAT4, EAAT5) peptide (PepT) transporters, Na^+^,dicarboxylate cotransporter (NaDC-1), creatine transporter (CreaT), Na^+^,myoinositol cotransporter (SMIT), phosphate carriers (NaPiIIa, NaPiIIb), Na^+^/K^+^-ATPase, albumin uptake	Lang et al., 2006, 2014. Lang et al., 2006; Lang and Stournaras, 2013.
Signaling molecules etc.,	nephrin, type A natriuretic peptide receptor (NPR-A), Ca^2+^-regulated heat-stable protein of apparent molecular mass 24 kDa (CRHSP24), adaptor precursor (APP) Fe65, NDRG1 and NDRG2, myosin-Vc, filamin C, microtubule-associated protein tau, Cyclin-dependent kinase inhibitor 1B (*p27*^Kip1^), huntingtin	McCaig et al., 2011; Ohashi et al., 2011; Lang and Stournaras, 2013; Sahin et al., 2013; Voelkl et al., 2015; Lang et al., 2018.

## SGK1 in the Regulation of Cell Function and Survival

SGK1 enhances cellular energy supply from glycolysis, at least in part by up-regulation of cellular glucose uptake and of the Na^+^/H^+^ exchanger with subsequent cytosolic alkalinization, which in turn enhances glycolytic flux (Kobayashi and Cohen, 1999; Rusai et al., 2010; Rotte et al., 2011).

SGK1 contributes to the regulation of embryonic and ischemic angiogenesis, an effect involving transcription factor NFκB, which in turn up-regulates vascular endothelial growth factor A (VEGF-A) thus promoting endothelial cell migration and tube formation (Catela et al., 2010; Zarrinpashneh et al., 2013).

In keeping with its role in cell survival and proliferation, copy number variation, as well as an increase in the expression and/or activity of SGK1, has been found in several human tumors (Brunet et al., 2001; Shelly and Herrera, 2002; Zhang et al., 2005; Heise et al., 2010; Schmidt et al., 2014; Lee et al., 2019). In agreement, SGK1 overexpression can confer resistance of cancer cells to chemotherapy and radiation (Sommer et al., 2013; Talarico et al., 2016). SGK1 is partially effective by enhancement of store-operated Ca^2+^ entry (SOCE), which is accomplished by the Ca^2+^ channel ORAI1 and its regulator STIM1 (Eylenstein et al., 2011, 2012; Borst et al., 2012; Schmidt et al., 2014). SOCE triggers cytosolic Ca^2+^ oscillations with subsequent depolymerization of the actin filaments and the decomposition of the actin skeleton is a prerequisite for cell proliferation (Schmid et al., 2012). The electrical driving force for Ca^2+^ entry is maintained by SGK1 sensitive K^+^ channels (Seebohm et al., 2005). Further, SGK1 supports cell proliferation and survival by inactivating the pro-apoptotic transcription factor FOXO3A/FKRHL1 (Amato et al., 2013; Sahin et al., 2013), by inhibition of GSK-3 leading to stimulation of β-catenin (Zhong et al., 2014), and by stimulation of IKKβ leading to activation of NFκB (Zhang et al., 2005). SGK1 also, activates Mouse Double Minutes 2 (MDM2)- an ubiquitin ligase inducing degradation of p53 (Amato et al., 2009). In addition, SGK1 can disrupt binding of SEK1 to JNK1 and MEKK1 (Kim et al., 2007) and enhances Ran binding protein (RanBP), which in turn interacts with microtubule organization and mitotic stability (Amato et al., 2013). Through RANBP, SGK1 stringently governs the nucleo-cytoplasmic shuttling of pre-miRNAs, a necessary condition for miRNA maturation, thus modulating the epigenome (Dattilo et al., 2017).

SGK1 can also modulate cellular motility (Caglayan et al., 2011; Schmidt et al., 2012; Zarrinpashneh et al., 2013; Walker-Allgaier et al., 2017; Liu et al., 2018). SGK1-dependent mechanisms contributing to migration include activation of NFκB with subsequent up-regulation of ORAI/STIM and thus of store-operated Ca^2+^ entry (Eylenstein et al., 2011; Walker-Allgaier et al., 2017). Moreover, SGK1 stimulates Na^+^,K^+^,2Cl^–^ cotransporters and Na^+^/H^+^ exchangers (Wang et al., 2005), carriers involved in cellular migration (Stock and Schwab, 2009; Delpire and Gagnon, 2018). Collectively, SGK1 can thus augment invasiveness and metastasis.

SGK1 expression is enhanced by TGF-β which is a stimulator of fibrosis (Akhurst and Hata, 2012; Voelkl et al., 2013; Guo et al., 2016). TGF-β-activated transcription factors include SMAD2 and SMAD3. SGK1 in turn augments TGF-β effects by inactivation of the ubiquitin ligase NEDD4L that normally triggers the degradation of SMAD2/3 (Gao et al., 2009; Xu et al., 2012). Signaling involved in the stimulation of fibrosis by SGK1 further includes pro-fibrotic transcription factor NFκB (Shih et al., 2011; Stone et al., 2011) and connective tissue growth factor (CTGF) (Das et al., 2012; Voelkl et al., 2012; Yang et al., 2012; Chilukoti et al., 2013; Tsai et al., 2014).

SGK1 stimulates tissue calcification, which is in part by up-regulation of osteo-/chondrogenic signaling including activation of the transcription factors MSX2 and CBFA1 (Voelkl et al., 2018). The effect again involves up-regulation of NFκB, a potent stimulator of vascular calcification (Zhao et al., 2012; Voelkl et al., 2018).

## SGK1 in the Regulation of Endometrial Function and Infertility

**FIGURE 1:**
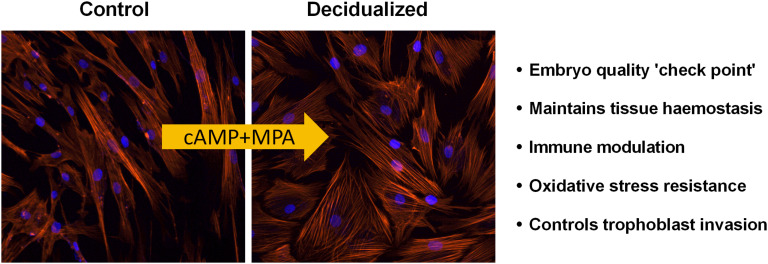
Decidualization of endometrial stromal cells: Endometrial decidualization, a process imperative for pregnancy in all species with invading embryos, is characterized by the transformation of stromal fibroblasts into secretory decidual cells. Decidual cells are *a priori* programmed to select against poor quality embryos. Modified and adapted from Alauddin et al. (2020) with permission.

The endometrium, the functional layer of the uterus acts as the site for embryo implantation and nutrient support for the growing conceptus (Aghajanova et al., 2008). Embryo implantation and fetal growth strictly depend on the receptive phenotype expressed by the endometrium during the implantation window (6 days post ovulatory phase) with subsequent differentiation of the stromal fibroblasts into specialized decidual cells (Gellersen and Brosens, 2003; Teklenburg et al., 2010; Lucas et al., 2013). The decidualization process is accompanied by transition of thin elongated stromal cells into an enlarged, rounded morphology and induction of the specific marker genes such as insulin-like growth factor-binding protein 1 (IGFBP-1) and prolactin (PRL) (Teklenburg et al., 2010; Kusama et al., 2013). This process is mediated by cyclic adenosine monophosphate (cAMP) signal transduction coupled with progesterone signaling (Brar et al., 1997; Gellersen and Brosens, 2003). It is also characterized by the influx of maternal natural killer (uNK) cells, vascular remodeling and transient oedema (Gellersen and Brosens, 2014). Decidual cells play an important task in embryo ‘check point’ or ‘quality control’ and pro-actively reject developmentally impaired blastocysts, as these cells are acutely accustomed to react to signals from unhealthy embryos (Salker et al., 2010). Decidual cells also aid in establishing functional feto-maternal interface, maintain tissue haemostasis, modulate immune cells and can control trophoblast invasion (Figure 1). Additionally, they exert cellular anti-oxidative defense against the reactive oxygen species (ROS) generated at the feto-maternal interface in addition to silencing of pro-apoptotic pathways (Dey et al., 2004; Kajihara et al., 2006; Leitao et al., 2010; Salker et al., 2012; Lou et al., 2016).

miRNAs constitute a family of short non-coding RNA molecules of 18–24 nucleotides in length that regulate gene expression at the post-transcriptional level, targeting sequences in the transcript 3′ untranslated regions (3′UTR) that are only partially complementary to the miRNA, causing a repression of protein synthesis (Bartel, 2009; Wahid et al., 2010; Gulyaeva and Kushlinskiy, 2016). miRNAs are expressed in most organisms and regulate the expression of genes which are involved in different biological processes including cell proliferation, embryo development, organ development and cell motility (Christopher et al., 2016). More than 2500 different mature miRNA species present in humans have been registered in the miRNA database (Juzenas et al., 2017; Kozomara et al., 2019). Overexpression and underexpression of miRNAs are associated with a variety of pathologies including: ectopic pregnancy, endometriosis and endometrial cancer (Galliano and Pellicer, 2014; Yanokura et al., 2015). In the human endometrium, several studies, including next generation sequencing have identified the expression of several miRNAs. Among those altered include members of the miR-200 family (Alowayed et al., 2016; Tamaru et al., 2020).

Furthermore, miRNAs can also be secreted by cells into the extracellular environment (Turchinovich et al., 2012). Extracellular miRNAs exist in association with proteins, bound to lipoproteins or can be packaged into membrane-bound extracellular vesicles (EVs) (Groot and Lee, 2020). Recently, a study suggested that EVs can serve as an important pathway for the transfer of genetic information at the maternal-fetal interface and potentially, could control gene expression in the recipient cells and alter its physiology (Kurian and Modi, 2019; Tan et al., 2020). Thus, miRNAs also play a crucial role in the regulation of the endometrial physiology and the implantation process of the embryo. Furthermore, miRNAs are emerging diagnostic markers and potential therapeutic tools for understanding implantation disorders. However, further research is needed before miRNAs can be used in clinical practice for identifying and treating implantation failure.

Failed interaction between the invading blastocyst and the endometrial surface coinciding along with an impaired maternal decidual response is thought to be a major cause for infertility and pregnancy loss (Koot et al., 2012; Zhang et al., 2013; Lou et al., 2016). With alarming increase in infertility cases affecting about 10% of couples both in developed and developing countries, investing in strategies to understand the endometrial function is of importance. Studies suggest uterine receptivity to the invading embryo and fetal survival depends on the regulation of key uterine regulators (Dey et al., 2004; Cha et al., 2012; Koot et al., 2012; Zhang et al., 2013).

SGK1 is one such key regulator that is believed to have a major role in regulating a myriad of physiological processes important in pregnancy success (Salker et al., 2011; Cha et al., 2012; Lou et al., 2016). The primary evidence to imply the role of SGK1 in endometrial function and fertility arose from a microarray screening experiment of mid luteal (LH +5 to +10) endometrial biopsies obtained from both fertile (control) and infertile women (Feroze-Zaidi et al., 2007). SGK1 was significantly elevated in response to an increase of progesterone levels during the mid-luteal phase of the cyclic endometrium in both luminal epithelial cells and then in decidualizing stroma (Feroze-Zaidi et al., 2007; Salker et al., 2011). Importantly, these studies showed that during the implantation window there must be a decline in SGK1 expression levels in luminal epithelium to infer endometrial receptivity (Feroze-Zaidi et al., 2007; Salker et al., 2011).

To define the exact role of SGK1 in implantation, the expression levels of SGK1 in the mid-secretory phase endometrial biopsies from subjects with proven live birth (fertile) or with recurrent/unexplained implantation failure were examined (Salker et al., 2011). This study showed that enhanced endometrial SGK1 activity was associated in women with unexplained infertility in comparison with fertile controls in the luminal epithelium (Salker et al., 2011; Lou et al., 2016). Further, to investigate if sustained activity of SGK1 in the endometrial epithelium interferes with embryo implantation, an expression vector encoding constitutively active SGK1 (SGK-S422D) was injected into the uterine lumen of wild type female mice (using a natural mating model). The results showed a complete abolishment of implantation sites in animals expressing constitutively active SGK1 (Salker et al., 2011).

SGK1 acts as a regulator of ENaC and several other ion channels. SGK1 directly regulates ENaC activity in the endometrium (Salker et al., 2016) and enhances its expression through inhibition of ubiquitin ligase NEDD 4-2 (Heise et al., 2010; Salker et al., 2011). ENaC detected in epithelial cells is a major modulator of uterine luminal fluid (ULF) secretion and reabsorption before embryo implantation, thereby promoting endometrium receptivity and implantation (Yang et al., 2004; Ruan et al., 2012; Zhang et al., 2013).

**FIGURE 2:**
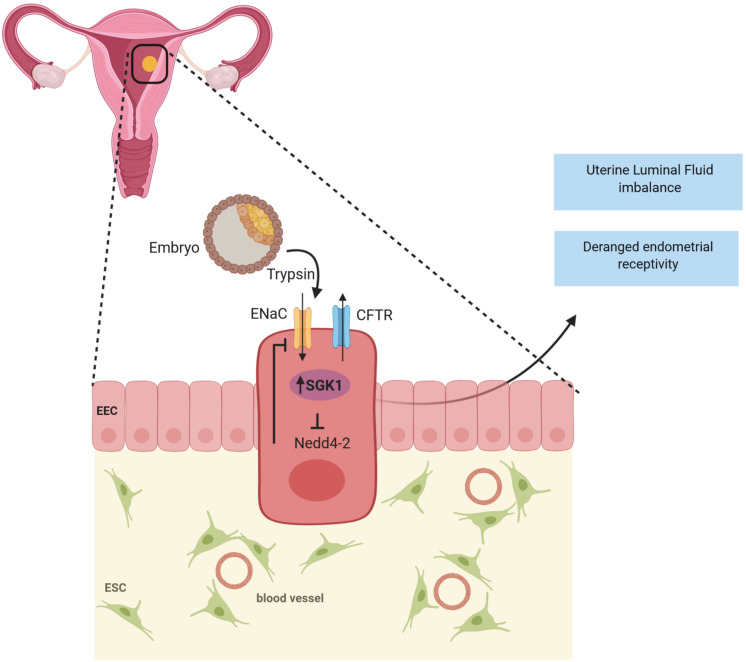
SGK1 in infertility: A transient decline of SGK1 activity in the endometrial epithelium promotes blastocyst apposition by regulating the activities of Epithelial sodium channel (ENaC) and Cystic fibrosis transmembrane conductance regulator (CFTR) to achieve appropriate uterine luminal fluid (ULF) secretion and absorption during the window of endometrial receptivity to confer successful implantation. Whereas, when expressed in high levels in the luminal epithelium, SGK1 is speculated to perturb uterine luminal fluid balance, attenuate endometrial receptivity and thereby resulting in implantation failure.

In addition to ENaC, elevated SGK1 levels also upregulates the cystic fibrosis transmembrane conductance regulator (CFTR) (Salker et al., 2011), which is an another vital factor that is associated with abnormal ULF accumulation and in *in vitro* fertilization (IVF) treatment failure (Yang et al., 2004; Chan et al., 2009; Salker et al., 2011; Lou et al., 2016). Therefore, it could be postulated that a sustained increase of SGK1 in endometrium epithelium could in fact perturb ULF (Figure 2) by causing premature uterine closure aiding in insufficient adhesion of the blastocyst to the uterine epithelium or creating a hostile environment which prevents implantation (Lou et al., 2016). Given the diverse roles of SGK1 in the regulation of ion channels, it remains to be determined which other channels are involved in regulating ULF and implantation. Furthermore, aberrant expression of SGK1 in the mouse luminal epithelium either suppressed or completely abolished the induction of key endometrial receptivity markers, such as leukemia inhibitory factor (Lif), heparin-binding EGF-like growth factor (Hbef) and homeobox protein Hox-A10 (Hoxa10) leading to conception delay and thus implantation failure (Fisher and Giudice, 2011; Salker et al., 2011; Lou et al., 2016). Therefore, it is conceivable that excessive SGK1 in the luminal epithelium of the endometrium could disturb the delicate ULF balance causing a hostile environment thus preventing embryo implantation as well as dysregulating (directly or indirectly) endometrial receptivity genes, which contributes to unexplained implantation failure in humans.

## SGK1, Obesity and Diabetes

Both obesity and diabetes are associated with reproductive failure (Dağ and Dilbaz, 2015; Thong et al., 2020). SGK1 participates in the development of obesity (Li et al., 2013), which is well known to cause insulin resistance and eventually leading to type 2 diabetes (Lang et al., 2009). SGK1 can promote the development of obesity by stimulation of the Na^+^ coupled glucose transporter mainly by SGLT1, which aids intestinal uptake of glucose (Jeyaraj et al., 2007). Rapid glucose absorption results in excessive insulin release and deposition of fat, with a following decrease of plasma glucose concentration, which prompts repeated glucose uptake and thus obesity (Bano, 2013). Further, overexpression of SGK1 enhances adipocyte differentiation (Di Pietro et al., 2010). The role of SGK1 in the development of obesity is emphasized by the finding that the I6C/E8CC/CT SGK1 variant is related to increased body weight (Busjahn et al., 2004) and is more prevalent in patients with type 2 diabetes than in individuals without a family history of diabetes (Schwab et al., 2008). This polymorphism which pre-disposes to obesity, is also associated with moderately enhanced blood pressure and hyperinsulinemia, which are all key factors in metabolic syndrome (Norlander et al., 2017).

In diabetes mellitus, excessive plasma glucose concentrations can upregulate intestinal SGK1 expression and thus SGK1-dependent stimulation of SGLT1, contributing to the maintenance of obesity (Dieter et al., 2004). A number of studies have proposed that abnormal glucose metabolism in the endometrium contributes to the increased risk of adverse pregnancy outcomes (Salker et al., 2017; Flannery et al., 2018; Raglan et al., 2020). It has now been reported, that SGLT1 is indeed expressed in the endometrium and controls glycogen accumulation essential for histiotrophic nutrition in pregnancy (Salker et al., 2017). Further, loss of Sglt1 compromised endometrial glycogen storage, resulting in a reduced litter size and low birth weight in mice. Comparative SGLT1 deficiency in the human endometrium (at implantation) predisposed for early pregnancy failure and obstetrical complications, including fetal growth restriction (Salker et al., 2017). In view of the present observations, particular caution is warranted in pre-conception or during pregnancy if SGLT1 inhibitors are considered for the treatment of diabetes (Kalra et al., 2015). Further, SGK1 inhibitors are useful, however, only, if they do not hinder the activity of the PKB/Akt isoforms, which would impede cellular glucose uptake and thus would be expected to exacerbate diabetic hyperglycemia.

Women beginning their pregnancies as either overweight [body mass index (BMI), 25–29.9 kg/m2] or obese (BMI ≥ 30 kg/m2) have an increased risk to deliver large for gestational age (LGA) babies (Ziauddeen et al., 2019). Placental nutrient transport is controlled by fetal, placental and maternal factors. It is well established that IGF-I, insulin and mTOR signaling stimulate placental amino acid transport (Rosario et al., 2011, 2015). All three factors can stimulate SGK1 (Park et al., 1999; Hall et al., 2012). Whether feedback mechanisms exist to perpetuate this pathway requires further elucidation. These studies will increase our understanding of the mechanisms linking maternal obesity and LGA at birth may facilitate the development of novel intervention strategies to alleviate excessive fetal overgrowth.

## SGK1 and Endometriosis

Endometriosis is a debilitating disease that affects 5–10% of women of reproductive age (González-Echevarría et al., 2019). Clinical manifestations of endometriosis include inflammation, chronic pelvic pain and infertility (Klemmt and Starzinski-Powitz, 2018). The pathogenesis of endometriosis has not been clearly defined though the current paradigm is thought to be *via* retrograde menstruation (Sourial et al., 2014). Endometriotic tissues are estrogen-dependent and are associated with enhanced aromatase expression and local estrogen production (Kitawaki et al., 2002). It is noteworthy to point out that hypomethylation of the Estrogen Receptor 2 (*ESR2)* promoter is associated with increased gene and protein levels in pathological tissues and an increased ‘inflammatory state’ (Xue et al., 2007).

Additionally, it has been demonstrated that SGK1 expression is significantly increased in human endometriotic lesions compared with healthy controls and that PGE2 mediated-inflammation perpetuates SGK1 activity (Lou et al., 2017). Oestradiol-sensitive overexpression of SGK1 by *ESR2* promoted endometriotic stromal cell survival by phosphorylating and inactivating the pro-apoptotic transcription factor, FOXO3a (Monsivais et al., 2016). These findings agree with the role of SGK1 in cell proliferation and survival. Additionally, endometrial SGK1 up-regulates the activities of Orai1 and SOCE, which triggers cell proliferation and migration, thereby perpetuating endometrial regeneration of abnormal tissue (Schmidt et al., 2014). This biological role of SGK1 in the endometrium has important bearings on implantation because of the frequency of infertility in women with endometriosis.

## SGK1 and Pregnancy Loss

Once the embryo has adhered to the luminal epithelium of the endometrium *via* ligand dependent interactions, the invading embryo is actively encapsulated by the underlying decidualized stromal cells. These highly specialized cells aid in defining healthy feto-maternal interface, desirable for embryo development (Lucas et al., 2013).

Although SGK1 expression and activity decline during the implantation window in the uterine epithelium, it is found to be highly induced in the decidualizing stroma underlying the epithelium. The decidua regulates trophoblast invasion and placenta formation (Dey et al., 2004). SGK1 levels are high in the early pregnancy decidua compared with normal menstrual endometrium upon stimulation of progesterone (Salker et al., 2011; Lou et al., 2016). Phospho-SGK1 levels are downregulated in the stromal cells in mid-secretory endometrium samples of women with recurrent pregnancy loss (>3 or more losses prior to 24 weeks of gestation), a finding directing to the possible protective role of SGK1 activity in the maternal decidual response (Salker et al., 2011).

**FIGURE 3:**
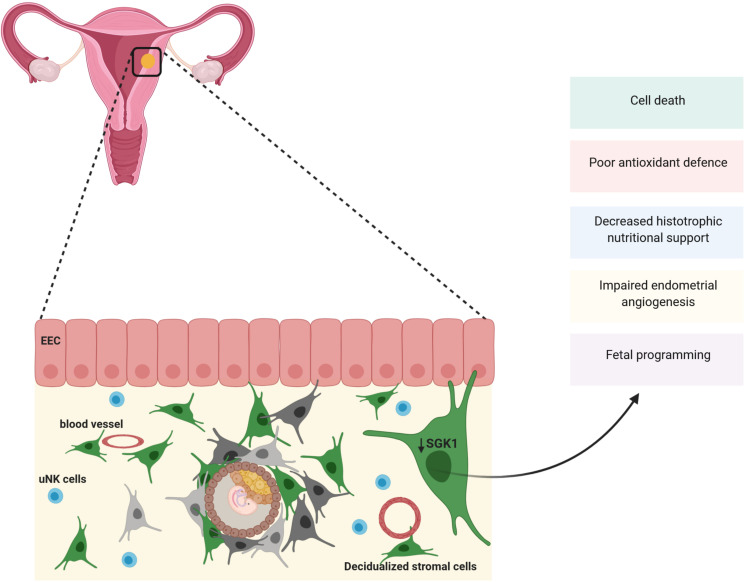
SGK1 and pregnancy loss: Expression of SGK1 rises in the decidual (stroma) compartment of the endometrium to sustain early pregnancy. SGK1 modulates cell differentiation, proliferation and survival of the decidua and provide with antioxidant defenses at the feto-maternal interface. Thus, when SGK1 levels fall in the decidual stroma it results in poor cell proliferation, reduction in antioxidant defense resulting in cell death and reduction in histotrophic nutritional support causing early pregnancy loss. Low maternal SGK1 levels could also possibly influence endometrial angiogenesis and fetal programming.

To further explore the role of maternal SGK1, these authors turned to the SGK1 knockout mouse model. The authors revealed that in pregnant *Sgk1 knockout* (*sgk1^–/–^)* mice, the uteri/implantation sites, showed evidence of uterine bleeding, deficiency in fetal growth and spontaneous (30%) fetal loss akin to human miscarriage (Fisher and Giudice, 2011; Salker et al., 2011). In line with this, *in vitro* SGK1 knockdown sensitizes decidual stromal cells to considerable oxidative stress at the utero-placental interface by deregulation of free radial scavengers (GPX3, SOD2, TXN, GLRX1 and PRDX2) subsequently inducing excessive intracellular ROS resulting in cell apoptosis at the maternal fetal interface (Salker et al., 2011; Figure 3).

As mentioned above, SGK1 supports cell proliferation and survival by regulating various membrane ion transporters as well as K^+^, Cl^–^ and Ca^2+^ channels (Lang et al., 2006). Specifically, SGK1 contributes to survival of endometrial stromal cells, which is essential for decidualization *via* phosphorylation and subsequent inactivation of pro-apoptotic FOXO3a (Lou et al., 2016; Monsivais et al., 2016). Thus, loss of SGK1 in stromal decidua represses stromal cell proliferation and survival required for decidualization, dysregulates intracellular ion homeostasis in differentiating cells and leads to oxidative cell death at the maternal-fetal interface causing pregnancy failure (Salker et al., 2011; Lou et al., 2016).

In summary, the different mechanistic impact on embryo implantation and pregnancy maintenance in the two distinct cellular compartments of the endometrium exerted by SGK1 makes it a key player in maintaining the functional reproductive axis. More precise understanding of the role of SGK1 in reproductive disorders mainly in infertility and miscarriage will provide potential promising opportunities for clinical intervention.

## SGK1 and Immune Cells in Pregnancy

The fetus has a genetic makeup derived from both paternal and maternal genes and is therefore a semi-allograft to the maternal immune system (Mor and Cardenas, 2010; Negishi et al., 2018). Intriguingly, the fetus evades immune rejection and is tolerated for the duration of pregnancy. This phenomenon was first described by Sir Peter Medawar in 1953 as ‘the paradox of pregnancy’ (Solano, 2019). The mechanism behind this remains a central question in reproductive immunology. During the decidual process, there is an influx of innate immune cells that is primarily populated by three leukocyte subpopulations, namely uNK cells, macrophages, and T cells. Antibody producing B cells are virtually absent (Suzuki et al., 1995; Bulmer and Lash, 2019; Solano, 2019).

The largest populations (10–15%) of decidual immune cells are the uNK cells (CD56^bright^CD16^–^) (Bulmer and Lash, 2019). They participate in coordinating important pregnancy events such as implantation and placentation (Sojka et al., 2019). uNK cells are highly localized to the areas surrounding the specialized decidual cells (Bulmer and Lash, 2019). Decidualization and the unique endocrine signals from decidua result in recruitment of NK cells from the periphery to the endometrium (Solano, 2019). Chemokines/cytokines such as IL-15, IL-7 and TGFβ-1 secreted by the uterine decidua attract NK cells to the endometrium and also stimulate their differentiation (Kauma et al., 1996; Rebecca et al., 2006). These factors are increased in the endometrium during the secretory phase and in early pregnancy. uNK cells secrete a plethora of cytokines such as granulocyte macrophage colony stimulating factor (GM-CSF), Interferon-γ (IFN-γ), IL-2, IL-10, leukemia inhibitory factor (LIF) and matrix metallopeptidase (MMP), suggesting that uNK cells may have functions related to the dialogue at the maternal-embryonic interface and subsequent development of the placenta (Kalkunte et al., 2009; Hara et al., 2016; Sojka et al., 2019). RNA sequencing and explant studies characterized uNK cells as highly pro-angiogenic cells that produce angiogenic factors such as members of the VEGF family, including placenta growth factor (PLGF) (Quenby et al., 2009; Gong et al., 2014). Mouse knock-out models devoid of uNK cells have shown that uNK cells not only participate in angiogenesis, but also regulate the structure of blood vessels in the uterus (Croy et al., 2003). Recently, it was shown that a subpopulation of endometrial stromal cells undergoes senescence during decidualization (Brighton et al., 2017). These senescent cells were then cleared by uNK cells *via* the perforin/granzyme B pathway (Brighton et al., 2017). Homeostasis of decidual cell senescence and uNK cell clearance is proposed to be essential for the establishment of a healthy window of implantation (Brighton et al., 2017; Lucas et al., 2020). Dysregulation in these processes may well contribute to reproductive failure. The role of uNK cells in pregnancy still remains unclear; however, this is one of the first reports of their critical activity within the non-pregnant uterus.

Besides NK cells, T cells are also a major cell population in the endometrium and decidua (Lissauer et al., 2017). It has been long proposed that maternal tolerance is established by a predominance of T helper type 2 (Th2) immunity over Th1 immunity during pregnancy (Th1/Th2 balance) (Sykes et al., 2012). SGK1 is a positive regulator of Th2 differentiation and a negative regulator of Th1 differentiation (Heikamp et al., 2014; Norton and Screaton, 2014). In murine CD4^+^ helper T cells, SGK1 can phosphorylate GSK-3β and block the degradation of β-catenin (Zhu et al., 2020). This, in turn, can inhibit downstream targets associated with the Th1 phenotype by increasing T cell factor 1 (TCF1) (Norton and Screaton, 2014). Moreover, SGK1 can also phosphorylate Nedd4-2, which inhibits the ubiquitination of JunB (Yang and Kumar, 2010). The stability of JunB contributes to IL-4 and GATA-3 expression, which is required for the Th2 cell lineage (Saito et al., 2010). Loss of SGK1 abolishes the inhibition of GSK-3β and Nedd4-2 (Di Cristofano, 2017), thus promoting the Th1 and inhibiting the Th2 phenotype. However, both Th1 and Th2 dominant immunity are observed in pregnancy complications (Feyaerts et al., 2017) therefore whether these pathways contribute to pregnancy maintenance needs further investigation.

Th17 cells and regulatory T (Treg) cells have been shown to play an important role in pregnancy (Figueiredo and Schumacher, 2016). SGK1 contributes to the regulation of inflammation and is decisive for the up-regulation of Th17 cells with subsequent release of the pro-inflammatory cytokines GM-CSF, TNF-α and IL-2 (Wu et al., 2013, 2018). SGK1 regulates IL-23 signaling by phosphorylating Foxo1 and reducing its nuclear exclusion, thus promoting IL-23 receptor expression (Wu et al., 2013). The pathogenic functions of Th17 cells are enhanced by the IL-23 receptor. Foxp3, a critical regulator of Treg cell development and function is also controlled by Foxo1 activity (Harada et al., 2010). Although much effort has been put in elucidating how the immune system contributes to pregnancy, particularly in mice, knowledge on early human pregnancy is limited.

Therefore, the simplistic notion that the Th1/Th2 balance is necessary for a healthy pregnancy is likely to be replaced with a complex interplay including different helper T cells (Th1/Th2/Th17/Treg axis). Therefore, future studies should explore the influence of different triggers on immune cells in the uterus before and during pregnancy and, how deregulation can lead to pregnancy complications.

One of the important components of the innate immune population are the gamma/delta (γδ) T cells (Mincheva-Nilsson, 2003). Earlier studies show that these cells play a pivotal role in maintaining homeostasis by clearing infections and also ensuring immune tolerance at the feto-maternal interface (Carding and Egan, 2002). Thus, these cells play both a regulatory and an effector role (Suzuki et al., 1995). Interestingly Mincheva-Nilsson et al., showed an extrathymic differentiation of γδ T cells in the decidua (Mincheva-Nilsson et al., 1997). This could help prime the maternal immune system to the growing fetus, as the first encounter and presentation of fetal antigens to the mother takes place in the decidua. Thus, the expansion and maturation of γδ T cells in the uterine decidua might be a possible mechanism aiding the maternal innate immune system to accept the pregnancy. Further, in celiac disease, SGK1 supports epithelial cell survival by inhibiting apoptosis induced by intraepithelial lymphocytes (IELs) (Szebeni et al., 2010). γδ T cells are present in abundance within the IELs cell population aiding in an epithelial defense mechanism (Cheroutre et al., 2011). It is tempting to speculate that overexpression of SGK1 in the uterine epithelium could drive enhanced epithelial cell protection and survival by inhibiting apoptotic signals from the uterine γδ T cells present. This altered epithelial cell death might have deranged effects on early phases of implantation, as physiological apoptosis of uterine epithelial cells is an essential checkpoint for successful blastocyst implantation in a process known as ‘displacement penetration’ (Joswig et al., 2003; Boeddeker and Hess, 2015).

The uterine immune milieu is believed to be strongly modulated by the crosstalk between the endometrial cells and the uterine immune cell population. The evidence of decidua acting as an extrathymic maturation site for γδ T cells and also controlling the homing and differentiation of uNK cells implies the importance of decidual microenvironment and the crosstalk between uterine cells in immunoregulation during pregnancy. Thus, it is reasonable to postulate that the aberrant levels of SGK1 in the endometrial compartments might have an unfavorable effect on the uterine immune cell populations. For instance, high levels of SGK1 in stromal cells causing impaired uterine decidua could also have adverse effect on differentiation of γδ T cells and uNK cells. Inefficient maturation of these immune cells at the decidua could lead to poor immune tolerance and improper vessel remodeling.

A better understanding of uterine SGK1 and immune modulation at the feto-maternal interface may help develop new diagnostic biomarkers and related targeted therapies for miscarriage and other obstetrical complications.

## SGK1 in Fetal Programming

The nutritional status of the mother is an important factor that affects fetal programming (Langley-Evans et al., 1994; Vehaskari et al., 2001; Woods et al., 2001; Lamireau et al., 2002; Manning et al., 2002; McMullen and Langley-Evans, 2005; Welham et al., 2005; Maria Pereira Pires et al., 2006; Pladys et al., 2006; Zambrano et al., 2006; Hocher, 2007; Dörsam et al., 2019; Rughani et al., 2020; Zhou et al., 2020). To investigate the impact of SGK1 on fetal programming and hypertension (blood pressure), Rexhepaj et al., mated wild-type (*sgk1^+/+^)* male mice with *sgk1^–/–^* female mice, and *sgk1^–/–^* males with *sgk1^+/+^* females, resulting in heterozygotic (sgk1^–/+^) offspring in both cases (Rexhepaj et al., 2008). During mating and pregnancy, the animals were subjected to low protein diet (8%). As a result prenatal protein restriction of wild type (*sgk1^+/+^)* maternal mice led to offspring with a slower weight gain and significantly higher blood pressure after birth (Rexhepaj et al., 2008).

Thus, maternal signals mediated by SGK1 play a decisive role in fetal programming of hypertension induced by prenatal protein restriction (Rexhepaj et al., 2008). In a rat model, the miR-200 family and their associated mRNAs from vascular specimens from the offspring of dams undernourished during gestation were dysregulated (Khorram et al., 2010). Interestingly, these changes in miR-200 were still prominent 1 year after birth. Bioinformatics analyses obtained from the miRbase server showed that there is a paired seed region for miR-200 on the SGK1 3′UTR. It is therefore tempting to speculate that miR-200 could directly modulate SGK1 expression as an upstream regulator thus, modifying the function of endothelial cells or angiogenic signaling during pregnancy.

SGK1 also participates in embryonic neoangiogenesis by phosphorylating its physiological substrate NDRG1 and modulating expression of angiogenic factor VEGFA *via* the NFκB signaling axis (Zygmunt et al., 2003; Zarrinpashneh et al., 2013; Lou et al., 2016). Also, SGK1 plays a role in vascular remodeling in angiogenesis by regulating the expression of notch-signaling genes and arterial markers Efnb2 and Nrp1 (Catela et al., 2010). Given this, loss of maternal SGK1 may contribute to a reduction in endometrial vessel development, thereby contributing to growth restriction (IGUR), thus a lower pup weight. However, to prove this conjecture more investigations are essential to better appreciate the function of endometrial SGK1 and angiogenesis. Thus, these studies propose that maternal signals facilitated by SGK1 have a substantial role to play in *in-utero* programming of fetal blood pressure induced by a restricted (maternal) protein diet during pregnancy.

## Future Perspective and Limitations

Despite years of research, we still lack clarity about how the endometrium supports implantation and the maintenance of an ensuing pregnancy. Though, several potential biomarkers of endometrial receptivity have been proposed, we still lack the in-depth theoretical knowledge to design targeted therapies for infertility. The numbers of infertility patients are staggering, ∼15% of couples cannot conceive and ∼30% of established pregnancies are lost, and these numbers are rising (Teklenburg et al., 2010). Thus, there is a crucial need for development in reproductive medicine both at the ‘bench and bedside’. Studies addressing human reproduction face many challenges, including the ethical unfeasibility of obtaining samples in normal gestation to study implantation and very early pregnancy in humans. Murine studies have been instrumental in shedding light into the intricacies of embryo implantation and pregnancy. However, the major hurdle is in translating the key molecules found from murine models to humans.

In the endometrium of humans and mice, SGK1 influences reproductive success by fine balancing expression and activity at the preimplantation stage and in the decidualizing stroma. SGK1s’ clinical implication in fertility and pregnancy loss makes it a potential target for therapeutic intervention. In particular, as the transient decline of SGK1 is beneficial for successful implantation, commercial SGK1 inhibitors, such as EMD638683, GSK650394, or the new SGK1 inhibitor, named SI113, might also be considered for treatment of infertility to aid patients who undergo IVF at the mid-secretory phase of the cycle or as a non-hormonal contraceptive.

Critically, most of the data presented are based on cell-lines and the fact that SGK1 deficiency (knock out), *in vivo*, has minimal consequences for normal homeostasis. These studies associate SGK1 activation to specific pathways are rather restricted and often susceptible to caveats, including the use of overexpression (e.g., plasmid DNA) systems and the obvious lack of a multi-cellular environment. Current and future efforts, notably utilizing genetically engineered or uterine specific mouse models, shRNA-based cell systems, organoids or organ-on-a-chip methodologies (Politi et al., 2004; Gnecco et al., 2017; Hibaoui and Feki, 2020) will facilitate progress and delineate the signaling molecules and pathways that are directly and specifically controlled by SGK1 in the endometrium and eventually provide more insight into the intricate maternal-fetal labyrinthine.

## Conclusion and Outlook

Globally, 90,000,000 couples (one in seven) have difficulties conceiving, and in excess of 30,000 IVF treatment cycles fail annually in Germany alone, usually because of embryo implantation failure and a further 15% of clinically recognized pregnancies go on to miscarry (European IVF-Monitoring Consortium (EIM) for the European Society of Human Reproduction and Embryology [ESHRE] et al., 2016). With delay in childbearing age and factors influencing fertility such as obesity, there will be a greater dependency on IVF. By 2027, the socio-economic impact for IVF (and its subsidiaries) is estimated to exceed $37.7 Billion USD (Research, 2020). These statistics are startling, but these numbers are likely to increase. Therefore, efforts have been made in this direction in recent years to try to realize novel methods for detection of biomarkers and drug targets.

This review has presented evidence regarding SGK1 as potential endometrial regulator. Dysregulated SGK1 is implicated in impaired endometrial receptivity, implantation failure, endometriosis and in fetal programming. In fact, there is currently great interest in identifying gene targets that can be used as markers for the early detection of endometrial disorders and fertility. However, the implications of the findings described throughout this review need to be confirmed in large clinical trials before they become a real approach in daily clinical practice for assessing embryonic and endometrial health, and thereby increasing pregnancy rates per transfer after IVF.

In summary, maternal SGK1 plays a decisive role in mediating initial interaction of the implanting blastocyst with the endometrium, helps to maintain functional feto-maternal interface for pregnancy survival and contributes to fetal programming. It is hoped that future studies will lead to improved diagnostic and predictive tools that will assist in patient stratification and ultimately, improved pregnancy outcomes.

## Author Contributions

All authors listed have made a substantial, direct and intellectual contribution to the work, and approved it for publication.

## Conflict of Interest

The authors declare that the research was conducted in the absence of any commercial or financial relationships that could be construed as a potential conflict of interest.
